# Two-Stage Sparse Recovery for Off-Grid Cascaded Channel Estimation in RIS-Assisted mmWave Systems

**DOI:** 10.3390/s26103240

**Published:** 2026-05-20

**Authors:** Zhiyu Han, Qiuyan Liu, Yanxia Cao, Yafeng Wang, Yifan Lin

**Affiliations:** 1 The Key Laboratory of Universal Wireless Communications, Ministry of Education, Beijing University of Posts and Telecommunications, Beijing 100876, China; hanzhiyu@bupt.edu.cn (Z.H.); yflin@bupt.edu.cn (Y.L.); 2 The China Unicom Research Institute, Beijing 100048, China; liuqy95@chinaunicom.cn (Q.L.); caoyx28@chinaunicom.cn (Y.C.)

**Keywords:** reconfigurable intelligent surface (RIS), channel estimation, linear bregman iteration, energy leakage, pilot overhead

## Abstract

Accurate cascaded channel estimation is crucial for unlocking the full potential of reconfigurable intelligent surface (RIS)-assisted millimeter-wave (mmWave) systems. While compressive sensing reduces pilot overhead, conventional estimators suffer from severe performance degradation due to off-grid leakage induced by the continuous nature of spatial angles. To address this issue, we propose a two-stage channel estimation framework that divides the estimation process into two sequential phases, namely support selection and amplitude recovery. Based on this framework, we design an algorithm termed TS-PO. In the first stage, a preconditioned linear Bregman iteration (PLBI) mechanism is employed to identify the true channel support. Subsequently, the second stage utilizes a localized orthogonal matching pursuit (OMP) refinement to accurately recover the physical channel gains. Simulation results demonstrate the effectiveness of the proposed TS-PO in suppressing off-grid energy leakage. Specifically, it effectively mitigates the estimation error floor, achieves high reconstruction accuracy under stringent pilot overhead constraints, and exhibits strong robustness in dense multipath environments.

## 1. Introduction

Millimeter-wave (mmWave) communication has been widely recognized as a pivotal technology for future wireless networks due to its abundant spectrum resources and capability to support ultra-high data rates [1,2]. However, mmWave signals suffer from severe free-space path loss and are highly susceptible to blockage, which severely limits their coverage [3,4]. Recently, reconfigurable intelligent surfaces (RISs) have emerged as a revolutionary paradigm to tackle this bottleneck [5,6,7]. By dynamically adjusting the phase shifts of massive low-cost passive reflecting elements, RISs can reconfigure the wireless propagation environment and establish virtual line-of-sight (LoS) links between the base station (BS) and the user [8,9,10]. Beyond conventional communication enhancement, the RIS has recently been leveraged to empower emerging 6G paradigms, such as secure Integrated Sensing and Communication (ISAC) networks, by providing optimized sensing illumination and disrupting malicious interceptions [11]. Despite its tremendous potential, realizing the performance gains of RISs strictly relies on accurate cascaded channel state information (CSI) acquisition [12,13,14,15]. Since the RIS elements are typically passive and lack signal processing capabilities, estimating the high-dimensional channel with limited pilot overhead poses a formidable challenge [16,17,18,19].

To acquire the CSI, traditional estimators based on Least Squares (LS) or Minimum Mean Square Error (MMSE) algorithms are straightforward but require an unaffordable pilot overhead that scales up with the massive number of RIS elements [20,21]. To alleviate this burden, a practical solution that has garnered attention is the semi-passive RIS architecture, wherein a limited number of active sensors are integrated to assist channel estimation [22,23,24,25]. While semi-passive designs effectively simplify the estimation protocol, fully passive RIS remains the most highly anticipated paradigm due to its lower hardware overhead. Consequently, advanced signal processing techniques have been extensively leveraged for passive RIS. Machine learning and deep neural networks have been introduced for channel estimation to reduce overhead [26,27,28,29,30]; however, they often require massive training datasets and lack mathematical interpretability. Alternatively, compressive sensing (CS) techniques have been widely adopted by exploiting the inherent sparsity of mmWave channels. Within the CS framework, sparse Bayesian learning (SBL) can achieve high estimation accuracy and mitigate off-grid effects through probabilistic inference [31]. Nevertheless, SBL inherently requires massive matrix inversions in each iteration, rendering it computationally prohibitive for large-scale RIS arrays. As highly efficient alternatives, greedy pursuit algorithms have established themselves as fundamental benchmark references in the literature. Specifically, the classical orthogonal matching pursuit (OMP) and its structured variants, such as Double-Structured OMP (DS-OMP), represent the benchmark approaches in this domain [32,33,34]. These conventional methods fundamentally operate by greedily identifying the channel support over a pre-discretized angular grid, which significantly reduces the computational complexity compared to probabilistic inference. However, despite their widespread adoption as theoretical and practical benchmarks, a critical challenge remains unresolved. Practical propagation paths are continuously distributed in the spatial domain. This continuous nature inevitably leads to a severe mismatch with the pre-discretized grid, causing prominent off-grid energy leakage. Furthermore, under such discretized modeling frameworks, this leakage induces strong correlations among atoms in the sensing matrix. Consequently, conventional on-grid estimators like OMP and DS-OMP are easily misled, inevitably suffering from incorrect support identification and severe error propagation. These coupled effects pose a fundamental bottleneck that conventional greedy estimators cannot adequately address through parameter tuning alone.

To address the aforementioned challenges, this paper proposes a robust two-stage cascaded channel estimation framework, termed TS-PO, for RIS-enhanced multi-user mmWave systems. Unlike conventional on-grid approaches that suffer from performance degradation under continuous angular domains, the proposed method achieves a balance between off-grid robustness and computational efficiency. The main contributions of this work are summarized as follows:

- The RIS-assisted multi-user cascaded channel estimation problem is formulated as a joint sparse recovery task. To handle the inherent off-grid leakage, a two-stage architecture is proposed to explicitly decouple support identification from coefficient estimation, thereby mitigating the coupling between basis mismatch and amplitude estimation.
- The TS-PO algorithm is developed, which integrates a preconditioned linear Bregman iteration (PLBI) for coarse screening with a localized greedy refinement for precise estimation. Specifically, the PLBI stage efficiently suppresses spurious responses to identify a reliable candidate support set with low complexity. Subsequently, a localized OMP is employed to refine the candidate support, followed by LS estimation within the reduced subspace to accurately recover channel gains and mitigate shrinkage bias.

Simulation results demonstrate that the proposed TS-PO algorithm achieves improved estimation accuracy under limited pilot overhead.

## 2. System Model and Transmission Scheme

### 2.1. Cascaded Channel Model

This paper investigates an RIS-enhanced mmWave multi-user system, where the base station (BS) has a uniform planar array (UPA) of $M=M_{1}\times M_{2}$ antennas, where $M_{1}$ and $M_{2}$ denote the number of horizontal and vertical elements of the UPA. The RIS is a programmable metasurface made up of *N* elements, which is arranged in the form of an $N_{1}\times N_{2}$ UPA with $N=N_{1}\times N_{2}$. In addition, there are *K* users, each equipped with a single antenna.

**Figure 1:**
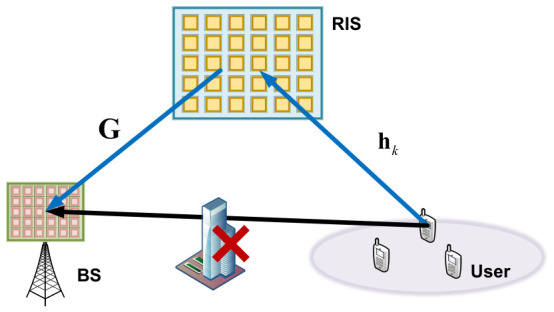
An RIS-enhanced multi-user wireless communication system.

As shown in Figure 1, we consider an uplink communication scenario. The signal is transmitted by the *k*-th user and reaches the BS through two links. One is the direct link from the *k*-th user to the BS where it is assumed that the direct link is obscured by obstacles and does not exist. The other is the reflective link which is divided into two parts, including the RIS-BS channel $G\in C^{M\times N}$ and the user-RIS channel $h_{k}\in C^{N}$. The reflective link can be specified as $G\Theta h_{k}\in C^{M}$ where $\Theta =diag\left(\theta \right)$ denotes the reflection matrix and $\theta ={\left[\theta_{1},\theta_{2},\cdots ,\theta_{N}\right]}^{T}\in C^{N}$. Further, the reflection link can be rewritten in terms of $G\Theta h_{k}=\;Gdiag\left(h_{k}\right)\theta$, and let $H_{k}=Gdiag\left(h_{k}\right)\in C^{M\times N}$, which is called the cascade channel of the *k*-th user.

Based on the Saleh–Valenzuela (SV) model, the RIS-BS channel can be modeled according to the sparsity of mmWave as [35]

(1) $$G=\sqrt{\frac{MN}{L_{G}}}\sum_{l_{G}=1}^{L_{G}}\beta_{l_{G}}a_{M}\left(\psi_{l_{G}}^{Gr},\varphi_{l_{G}}^{Gr}\right)a_{N}^{H}\left(\psi_{l_{G}}^{Gt},\varphi_{l_{G}}^{Gt}\right),$$

where $L_{G}$ represents the number of propagation paths in the RIS-BS channel. $\beta_{l_{G}}$ denotes the complex path gain of the $l_{G}$-th path. The variables $\psi_{l_{G}}^{Gt}$ and $\varphi_{l_{G}}^{Gt}$ are the azimuth and elevation angles of departure (AoDs) at the RIS, while $\psi_{l_{G}}^{Gr}$ and $\varphi_{l_{G}}^{Gr}$ represent the azimuth and elevation angles of arrival (AoAs) at the BS, respectively. Additionally, $a_{X}\left(\cdot \right)$ denotes the steering vector corresponding to the antenna arrays at the BS or the RIS. In this paper, the UPA steering vector with the number of antennas $N=N_{1}\times N_{2}$ is denoted by

(2) $$a_{N}\left(\psi ,\varphi \right)=\frac{1}{\sqrt{N}}\left[e^{-j2\pi d\sin\left(\psi \right)\cos\left(\varphi \right)n_{1}/\lambda }\right]⊗\left[e^{-j2\pi d\sin\left(\varphi \right)n_{2}/\lambda }\right]\in C^{N},$$

where $d=\frac{\lambda }{2}$ and λ is the carrier wavelength.

In the same way, the channel from the *k*-th user to the RIS can be expressed as

(3) $$h_{k}=\sqrt{\frac{N}{L_{h,k}}}\sum_{l_{h,k}=1}^{L_{h,k}}\beta_{l_{h,k}}a_{N}\left(\psi_{l_{h,k}}^{h,k},\varphi_{l_{h,k}}^{h,k}\right),$$

where $L_{h,k}$ denotes the number of paths of the user-RIS channel and $\beta_{l_{h,k}}$ denotes the complex path gain from the *k*-th user to the RIS. $\psi_{l_{h,k}}^{h,k}$ and $\varphi_{l_{h,k}}^{h,k}$ denote the horizontal and elevation angles of the AoA at RIS.

The cascaded channel $H_{k}$ can be rewritten in the virtual angular domain as

(4) $$H_{k}=V_{M}{\tilde{H}}_{k}V_{N}^{H},$$

where $V_{M}\in C^{M\times M}$ and $V_{N}\in C^{N\times N}$ are dictionary unitary matrices at the BS and the RIS. ${\tilde{H}}_{k}\in C^{M\times N}$ denotes the angular cascaded channel. Because there is limited scattering between RIS and BS, the matrix ${\tilde{H}}_{k}$ is sparse and contains only a small number of nonzero elements.

### 2.2. Transmission Model and Protocol

In this paper, we consider a time-division duplexing (TDD) system, where the uplink and downlink channels are assumed to be reciprocal. Thus, the downlink CSI can be effectively obtained by estimating the uplink channel.

**Figure 2:**
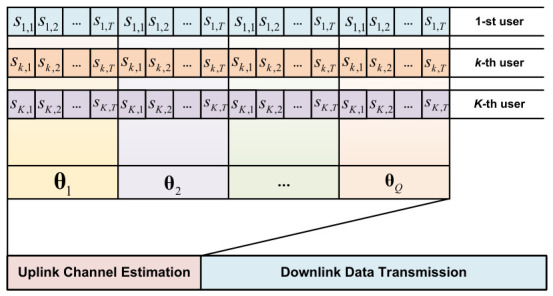
The proposed multi-subframe pilot transmission protocol for uplink cascaded channel estimation.

To recover the cascaded channel $H_{k}$ under limited pilot overhead, we propose a multi-subframe pilot transmission protocol, as illustrated in Figure 2. The uplink channel estimation phase consists of *Q* subframes, and each subframe contains *T* time slots (T≥K). During each subframe, the reflection coefficients of the RIS remain constant, while they vary across different subframes. To effectively separate the superimposed signals from multiple users at the BS, the *K* users transmit mutually orthogonal pilot sequences. Let $s_{k}={\left[s_{k,1},s_{k,2},\ldots ,s_{k,T}\right]}^{T}\in C^{T}$ denote the pilot sequence transmitted by the *k*-th user, which satisfies $s_{k_{1}}^{H}s_{k_{2}}=0$ for $k_{1}\neq k_{2}$, and $s_{k}^{H}s_{k}=PT$, where *P* is the transmission power.

Assuming that the direct links between the users and the BS are obstructed by blockages, the received signal $Y_{q}\in C^{M\times T}$ at the BS during the *q*-th subframe can be expressed as

(5) $$\begin{array}{cc} Y_{q} & =\sum_{k=1}^{K}G\mathrm{diag}\left(\theta_{q}\right)h_{k}s_{k}^{H}+W_{k,q}\\ \; & =\sum_{k=1}^{K}H_{k}\theta_{q}s_{k}^{H}+W_{k,q}, \end{array}$$

where $\theta_{q}\in C^{N\times 1}$ is the RIS reflection vector in the *q*-th subframe, and $W_{k,q}\in C^{M\times T}$ is the additive white Gaussian noise (AWGN) matrix, whose entries follow independent circularly symmetric complex Gaussian (CSCG) distributions with zero mean and variance $\sigma^{2}$.

By exploiting the orthogonality of the pilot sequences, the BS can perfectly decouple the signals of different users. Specifically, by right-multiplying $Y_{q}$ by $s_{k}$, the equivalent received signal vector $y_{k,q}\in C^{M\times 1}$ for the *k*-th user is given by

(6) $$y_{k,q}=\frac{1}{PT}Y_{q}s_{k}=H_{k}\theta_{q}+w_{k,q},$$

where $w_{k,q}=\frac{1}{PT}W_{k,q}s_{k}\in C^{M\times 1}$ is the equivalent noise vector.

We aggregate the received signals over all *Q* subframes. Let ${\tilde{Y}}_{k}=\left[y_{k,1},y_{k,2},\ldots ,y_{k,Q}\right]\in C^{M\times Q}$ be the overall signal matrix, $\Theta =\left[\theta_{1},\theta_{2},\ldots ,\theta_{Q}\right]\in C^{N\times Q}$ be the global RIS reflection matrix, and ${\tilde{W}}_{k}=\left[w_{k,1},w_{k,2},\ldots ,w_{k,Q}\right]\in C^{M\times Q}$. Thus, the aggregated signal in (6) can be rewritten in a compact matrix form as

(7) $${\tilde{Y}}_{k}=H_{k}\Theta +{\tilde{W}}_{k}.$$

## 3. Proposed TS-PO Channel Estimation Algorithm

In this section, the channel estimation problem is first formulated under a compressive sensing (CS) framework. To exploit the robustness of iterative reconstruction and the accuracy of greedy pursuit, a hybrid channel estimation scheme is developed by integrating preconditioned linear Bregman iteration (PLBI) with orthogonal matching pursuit (OMP). Specifically, PLBI is employed for coarse support detection, while OMP refines the support set and estimates the associated channel gains.

### 3.1. Problem Formulation and Motivation

Substituting (4) into (7) yields

(8) $${\tilde{Y}}_{k}^{H}=\Theta^{H}H_{k}^{H}+{\tilde{W}}_{k}^{H}=\Theta^{H}V_{N}{\tilde{H}}_{k}^{H}V_{M}^{H}+{\tilde{W}}_{k}^{H},$$

Based on the transmission protocol described in Section 2.2, (8) can be vectorized as

(9) $${\tilde{y}}_{k}=\left(V_{M}^{*}⊗\Theta^{H}V_{N}\right){\tilde{h}}_{k},$$

where ${\tilde{y}}_{k}=vec\left({\tilde{Y}}_{k}^{H}\right)$ and ${\tilde{h}}_{k}=vec\left({\tilde{H}}_{k}\right)$.

Based on (9), stacking the received signal matrices of different user yields

(10) $$\bar{Y}=A\bar{H},$$

where $\bar{Y}=\left[{\tilde{y}}_{1},{\tilde{y}}_{2},\cdots ,{\tilde{y}}_{K}\right]\in C^{MQ\times K}$, $A=V_{M}^{*}⊗\Theta^{H}V_{N}\in C^{MQ\times MN}$, and $\bar{H}=\left[{\tilde{h}}_{1},{\tilde{h}}_{2},\cdots ,{\tilde{h}}_{K}\right]\in C^{MN\times K}$.

In the RIS-enhanced mmWave multi-user system, the channel estimation problem can be modeled as a multiple-measurement-vector (MMV) CS problem.

Conventional greedy estimators, such as OMP, predominantly assume that the physical propagation paths align perfectly with the pre-discretized spatial grid. However, in practical mmWave deployments, the spatial angles are continuously distributed. This inherent basis mismatch leads to severe off-grid leakage, which destroys the sparsity of the channel in the virtual angular domain. Furthermore, the highly correlated columns of the sensing matrix cause severe coherent interference, inevitably misleading conventional algorithms to identify incorrect support sets. To fundamentally overcome these limitations, we propose a robust two-stage estimator, termed TS-PO, which decouples the estimation task by synergizing the preconditioned linear Bregman iteration (PLBI) with a localized OMP.

### 3.2. Stage I: Global Coarse Screening via PLBI

To effectively mitigate the off-grid leakage and suppress coherent interference, the first stage of the proposed TS-PO employs a threshold-based coarse screening mechanism. Since we are jointly estimating the cascaded channels for *K* users, the received signals are aggregated into an MMV model. Instead of directly solving the NP-hard $\ell_{0}$-norm minimization problem, we formulate it as an unconstrained $\ell_{1}$-norm regularized matrix optimization:

(11) $$\hat{\bar{H}}\;=\;\underset{\bar{H}}{\arg\min}\mu ||\bar{H}|{|}_{1}+\frac{1}{2}||\bar{Y}-A\bar{H}|{|}_{F}^{2},$$

where μ is the regularization parameter, $||\cdot |{|}_{F}$ denotes the Frobenius norm, and $||H|{|}_{1}$ represents the element-wise $\ell_{1}$-norm of the complex matrix.

While the classical linearized Bregman iteration (LBI) is efficient for single-measurement-vector (SMV) problems, its direct extension to the multi-user MMV setting is nontrivial [36]. More importantly, its convergence behavior is highly sensitive to the condition number of the sensing matrix A.

In RIS-enhanced mmWave systems, the continuously distributed angles leads to severe column correlations in A, which significantly deteriorates its condition number. This issue not only slows down convergence but also undermines the stability of iterative sparse recovery algorithms.

To address this fundamental limitation, it is necessary to incorporate a conditioning mechanism that can regularize the sensing matrix and stabilize the iterative updates. Motivated by this requirement, we adopt a PLBI scheme [37], where a preconditioning matrix ${\left(AA^{H}\right)}^{-1/2}$ is introduced to normalize the effective condition number.

By integrating this mechanism into the MMV formulation, the proposed approach enables stable and efficient sparse recovery under severe off-grid conditions. Applying the linearized Bregman divergence to the resulting preconditioned system yields the following matrix-form update equations:

(12) $$\left\{\begin{array}{c} U^{p+1}=U^{p}+A_{c}^{H}\left(\bar{Y}-A{\bar{H}}^{p}\right)\\ {\bar{H}}^{p+1}=\alpha {\mathrm{soft}}_{\mu }\left(U^{p+1}\right) \end{array}\right.,$$

where $A_{c}={\left(AA^{H}\right)}^{-1/2}A$, *p* denotes the iteration index, α is the step size, and U is the auxiliary dual matrix. The optimal soft-thresholding operator ${\mathrm{soft}}_{\mu }\left(\cdot \right)$ is applied element-wise to the matrix, defined as

(13) $$\begin{array}{cc} {\mathrm{soft}}_{\mu }\left(U\right) & =\frac{\max\left(\left|U_{r,c}\right|-\mu ,0\right)}{\max\left(\left|U_{r,c}\right|-\mu ,0\right)+\mu }U_{r,c}\\ \; & =\left\{\begin{array}{cc} \frac{U_{r,c}}{\left|U_{r,c}\right|}\left(\left|U_{r,c}\right|-\mu \right), & \left|U_{r,c}\right|>\mu \\ 0, & \left|U_{r,c}\right|⩽\mu \end{array}\right., \end{array}$$

where $U_{r,c}$ is the element of row *r* and column *c* in the complex matrix U, and $\left|U_{r,c}\right|$ is the absolute value of $U_{r,c}$.

This mechanism effectively acts as a robust spatial filter by suppressing spurious peaks induced by off-grid leakage while preserving the support indices of the true dominant paths. Consequently, Stage I outputs a highly reliable candidate support set for all users without performing tracking algorithms over the massive global dictionary.

### 3.3. Stage II: Support Refinement and Accurate Recovery

Although the PLBI scheme effectively sieves out the coherent interference, the soft-thresholding operation inevitably introduces amplitude shrinkage, leading to biased channel gain estimates. To achieve accurate signal recovery, the second stage employs a localized OMP refinement [38].

Let Λ denote the coarse candidate support set identified in Stage I. By extracting the associated columns, we construct a drastically reduced-dimensional sub-dictionary $A_{\Lambda }$. We initialize the local active support set as $\Gamma_{0}=Ø$ and the residual vector as $r_{0}=\tilde{y}$. At the *i*-th iteration, the algorithm identifies the optimal column index $j^{⋆}$ that exhibits the highest correlation with the current residual:

(14) $$j^{⋆}=\arg\max_{j\in \Lambda \setminus \Gamma_{i-1}}\left|a_{j}^{H}r_{i-1}\right|,$$

where $a_{j}$ represents the *j*-th column of $A_{\Lambda }$. The support set is then augmented by $\Gamma_{i}=\Gamma_{i-1}\cup \left\{j^{⋆}\right\}$.

To eliminate the amplitude shrinkage effect, the exact channel gains over the selected active paths are estimated via the Least Squares (LS) projection onto the subspace spanned by $A_{\Gamma_{i}}$:

(15) $${\hat{h}}_{\Gamma_{i}}={\left(A_{\Gamma_{i}}^{H}A_{\Gamma_{i}}\right)}^{-1}A_{\Gamma_{i}}^{H}\tilde{y}.$$

The residual is updated as $r_{i}=\tilde{y}-A_{\Gamma_{i}}{\hat{h}}_{\Gamma_{i}}$. Finally, the local indices within $\Gamma_{i}$ are mapped back to the global angular domain to formulate the ultimate sparse channel estimate $\hat{\tilde{h}}$.

The complete procedure of the proposed TS-PO estimator, which integrates the global coarse screening and the localized support refinement, is summarized in Algorithm 1.

**Remark** **1.** *In Algorithm 1, the physical sparsity level L is assumed to be known as the stopping criterion for the Stage II refinement. This assumption aligns with standard comparative benchmarks in the existing literature to ensure a fair evaluation of the ultimate support recovery capabilities. However, in practical deployments where the exact number of paths is unknown a priori, the proposed TS-PO can be seamlessly adapted. Specifically, the PLBI coarse screening in Stage I operates completely independently of L, as it identifies the candidate set based solely on a gradient soft-thresholding mechanism. For Stage II, the algorithm can easily replace the fixed-iteration stopping rule with a residual energy thresholding criterion. The iterative greedy search can be adaptively terminated when the energy of the residual falls below a predefined noise-dependent threshold (i.e., $||r_{k,i}|{|}_{2}^{2}\leq \epsilon$, where ϵ is strictly determined by the noise variance $\sigma^{2}$).*

### 3.4. Computational Complexity Analysis

In this subsection, we evaluate the computational complexity of the proposed TS-PO architecture and compare it with the conventional OMP algorithm. To facilitate the analysis, we denote the equivalent dimensions of the sensing matrix A as $\tilde{M}=MQ$ and $\tilde{N}=MN$, where $\tilde{N}$ represents the number of grid points in the massive global spatial dictionary. The sparsity level is denoted as $L=L_{G}\times L_{h,k}$.

The conventional OMP algorithm incurs a heavy computational burden during each iteration. It first requires calculating inner products across the massive global dictionary of size $\tilde{N}$. Additionally, it performs pseudo-inverse operations to update the signal support. Consequently, the total computational complexity for estimating *K* users is $O(KL\tilde{M}\tilde{N}+KL^{3}\tilde{M})$. As the dimensions of the RIS array scale up in 6G applications, the massive size of $\tilde{N}$ causes the global exhaustive correlation search to become a severe bottleneck, leading to unacceptable hardware latency and numerical instability. In contrast, the proposed TS-PO framework alleviates the prohibitive computational bottleneck associated with the massive global dictionary through a robust two-stage design.

- **Stage I** (PLBI Coarse Screening): The complexity is dominated by the linear matrix multiplications $A\bar{H}$ and $A_{c}^{H}\left(\bar{Y}-A\bar{H}\right)$ during the global gradient update. The complexity for this stage is $O(I_{iter}\tilde{M}\tilde{N}K)$, where $I_{iter}$ is the number of Bregman iterations. It is worth noting that this stage is completely free of any matrix inversion operations.
- **Stage II** (Localized OMP Refinement): Since the global dictionary $\tilde{N}$ has been effectively truncated into a small subspace with size *C* ($C\ll \tilde{N}$) by the optimal soft-thresholding mechanism, the local greedy search and pseudo-inverse only require a complexity of $O(KL\tilde{M}C+KL^{3}\tilde{M})$.

Therefore, the overall computational complexity of the proposed scheme is $O(I_{iter}\tilde{M}\tilde{N}K+KL\tilde{M}C+KL^{3}\tilde{M})$.
**Algorithm 1** Proposed TS-PO Estimator**Input:** Aggregated received signal matrix $\bar{Y}$, sensing matrix A, gradient step size α, threshold parameter μ, physical sparsity level $L=L_{G}\times L_{h}$, candidate set size *C*.**Output:** Estimated cascaded channel matrix ${\hat{H}}_{k},\forall k$.  1:**% Stage I: Global Coarse Screening via PLBI**  2:**Initialize: **$U^{\left(0\right)}=0$, ${\bar{H}}^{\left(0\right)}=0$, iteration index p=0.  3:Compute the preconditioning matrix $A_{c}={\left(AA^{H}\right)}^{-1}$.  4:**repeat**  5:    Update the auxiliary dual matrix:           $U^{\left(p+1\right)}=U^{\left(p\right)}+A_{c}^{H}\left(\bar{Y}-A{\bar{H}}^{\left(p\right)}\right)$;  6:    Update the channel matrix via element-wise soft-thresholding:           ${\bar{H}}^{\left(p+1\right)}=\alpha {\mathrm{soft}}_{\mu }\left(U^{\left(p+1\right)}\right)$;  7:    p←p+1;  8:**until** Convergence criterion is met or maximum iterations reached.  9:**% Stage II: Localized Support Refinement and Debiasing**10:**Initialize: **${\hat{H}}_{k}=0,\forall k$.11:**for** each user k=1 **to** *K* **do**12:    Extract the *k*-th user’s coarse estimate ${\tilde{h}}_{k}$ and received signal ${\tilde{y}}_{k}$.13:    Identify the user-specific candidate support set $\Lambda_{k}$ from the *C* dominant peaks of ${\tilde{h}}_{k}$.14:    Extract the reduced-dimensional sub-dictionary $A_{\Lambda_{k}}$.15:    **Initialize OMP:** Local active support $\Gamma_{k,0}=Ø$, residual $r_{k,0}={\tilde{y}}_{k}$.16:    **for** i=1 **to** *L* **do**17:        Identify the optimal local index with the maximum correlation:               $j^{⋆}=\arg\max_{j\in \Lambda_{k}\setminus \Gamma_{k,i-1}}\left|a_{j}^{H}r_{k,i-1}\right|$;18:        Augment the user-specific active support set: $\Gamma_{k,i}=\Gamma_{k,i-1}\cup \left\{j^{⋆}\right\}$;19:        Perform LS channel gain estimation:               ${\hat{h}}_{\Gamma_{k,i}}={\left(A_{\Gamma_{k,i}}^{H}A_{\Gamma_{k,i}}\right)}^{-1}A_{\Gamma_{k,i}}^{H}{\tilde{y}}_{k}$;20:        Update the orthogonal residual:               $r_{k,i}={\tilde{y}}_{k}-A_{\Gamma_{k,i}}{\hat{h}}_{\Gamma_{k,i}}$;21:    **end for**22:    Map the local estimate ${\hat{h}}_{\Gamma_{k,L_{\mathrm{paths}}}}$ back to the global dimensions to form the *k*-th column of ${\hat{\tilde{H}}}_{k}$.23:    ${\hat{H}}_{k}=U_{M}{\hat{\tilde{H}}}_{k}U_{N}^{H},\forall k$24:**end for**

Despite involving an $O(L^{3})$ pseudo-inverse operation, the TS-PO algorithm significantly reduces the overall computational burden compared to conventional greedy methods in large-scale systems. This efficiency is achieved by fundamentally circumventing exhaustive high-dimensional correlations across the massive global dictionary. Specifically, Stage I relies solely on matrix multiplications to efficiently screen the candidate support. Subsequently, Stage II restricts the greedy search and matrix inversion strictly to a localized candidate subspace. Due to the severe free-space path loss and limited scattering characteristics inherent in mmWave frequencies, the physical number of cascaded paths *L* is intrinsically sparse and bounded [39]. Consequently, the dimension of the matrix inversion remains exceptionally small, and the local search space *C* is drastically smaller than the massive global dictionary dimension $\tilde{N}$ ($C\ll \tilde{N}$). The proposed architecture circumvents computational bottlenecks by reducing the global exhaustive search to a low-dimensional local refinement, thereby ensuring scalability for large-scale RIS systems.

## 4. Simulation Results

In this section, we evaluate the performance of the proposed TS-PO algorithm through numerical simulations. We consider that the number of BS antennas is set to M=64 ($M_{1}=8$, $M_{2}=8$), and the number of RIS reflecting elements is N=256 ($N_{1}=16$, $N_{2}=16$). The system serves K=3 single-antenna users simultaneously. To simulate a realistic physical propagation environment, the spatial angles of all paths are continuously generated and uniformly distributed over $\left[-\frac{\pi }{2},\frac{\pi }{2}\right)$. In our simulations, the elements of the RIS reflection matrix are randomly and uniformly drawn from $\left\{-\frac{1}{\sqrt{N}},\frac{1}{\sqrt{N}}\right\}$ across all the subframes. $\left|\beta_{l_{G}}\right|=10^{-3}d_{RB}^{-2.2}$, where $d_{RB}$ denotes the distance between the RIS and BS and is assumed to be $d_{RB}=10$ m. $\left|\beta_{l_{h,k}}\right|=10^{-3}d_{UR}^{-2.8}$, where $d_{UR}$ denotes the distance between the user and RIS and is assumed to be $d_{UR}=100$ m [40]. Specifically, we use the normalized mean square error (NMSE) as the performance metric [39], which is given by

(16) $$NMSE=E\frac{||\hat{H}-H|{|}_{F}^{2}}{||H|{|}_{F}^{2}}.$$

Unless otherwise specified, the default system parameters are set as follows: the number of cascaded paths is $L_{G}=8$ and $L_{h,k}=5$, the pilot overhead is Q=32, and the signal-to-noise ratio (SNR) is fixed at 15 dB. The values of these simulation parameters are chosen to be consistent with typical practical settings in mmWave massive MIMO systems [39] and recent RIS-assisted channel estimation research [16,34]. Specifically, the number of propagation paths is set to accurately reflect the inherent sparsity of the mmWave channel, while the chosen pilot overhead ensures a rigorous evaluation of the algorithms under realistic low-latency constraints.

Regarding the algorithmic parameters for the proposed PLBI-based coarse screening stage, the step size is set to α=1 and the candidate support size is fixed as C=200. While the physical system parameters are fixed across all simulations to ensure a fair comparison, the regularization parameter μ plays a pivotal role in balancing interference suppression and signal preservation. Consequently, it is essential to determine its optimal value via empirical simulations.

**Figure 3:**
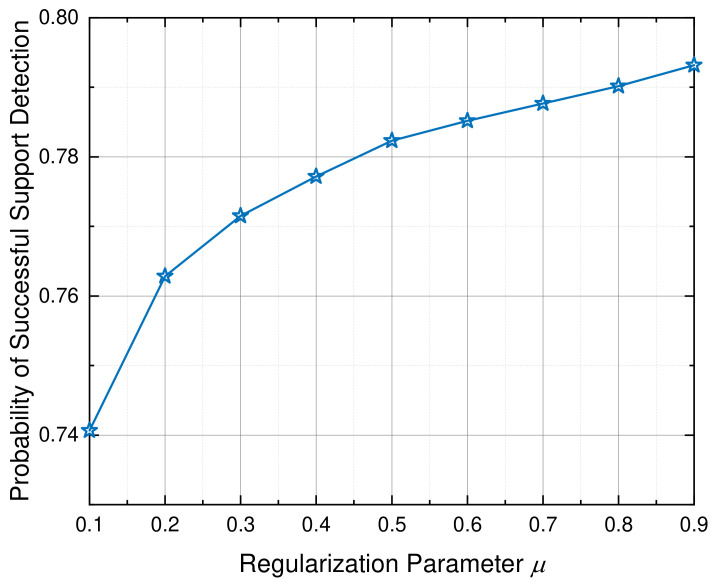
Probability of successful support detection versus the regularization parameter μ in the first stage.

Figure 3 illustrates the probability of successful support detection in Stage I as a function of μ. Specifically, this probability is defined as the ratio of the number of true paths successfully included in the candidate set to the total number of physical paths. As observed, the detection performance improves rapidly when μ increases from 0.1 to 0.4. This is because a larger threshold effectively suppresses spurious peaks caused by off-grid leakage and dense multipath interference. It is worth noting that while the detection probability continues to marginally increase and saturate for μ>0.5, an excessively large μ requires significantly more Bregman iterations to accumulate sufficient gradients, which leads to a severe increase in computational latency. Therefore, setting μ=0.4 corresponds to the optimal knee point of the curve, striking an excellent balance between robust support screening accuracy and computational efficiency.

With the optimal algorithmic parameters established, we now proceed to comprehensively evaluate the proposed TS-PO framework. We compare it with other state-of-the-art channel estimation schemes, including the conventional OMP [38], the structured DS-OMP [34], and the theoretical Oracle OMP bound where the supports of all sparse channels are assumed to be perfectly known. Although operating on the discretized grid, this Oracle estimator utilizes perfect a priori support knowledge, thereby completely circumventing support selection errors and error propagation induced by off-grid leakage. Consequently, it provides a fundamental lower bound for on-grid $\ell_{0}$-norm minimization. The performance gap between practical algorithms and this Oracle bound allows us to explicitly quantify the degree of leakage suppression achieved by the TS-PO. All subsequent simulation results are averaged over 500 independent Monte Carlo realizations.

**Figure 4:**
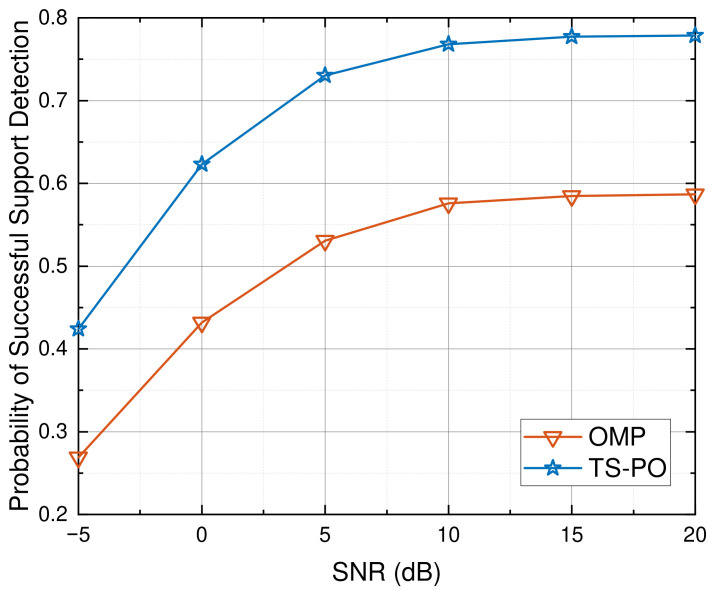
Probability of successful support detection versus varying SNR.

Since accurate overall channel estimation intrinsically depends on correct support identification, we begin our comparative analysis by investigating the support recovery capabilities of the algorithms. This investigation reveals the fundamental mechanism behind the proposed two-stage architecture. Figure 4 evaluates the probability of successful support detection for different algorithms versus SNR. As illustrated, the detection probability of all schemes generally improves with an increase in SNR. However, the OMP algorithm exhibits a relatively poor detection rate, particularly in the low-SNR regime, and saturates prematurely in the high-SNR regime. This limitation arises because OMP is highly susceptible to the coupled effects of off-grid leakage and severe dictionary coherence. Under noise interference, these coupled effects frequently mislead the greedy search, resulting in incorrect support selection. In contrast, the proposed TS-PO framework achieves a substantially higher probability of successful support detection across the entire tested SNR range. This significant superiority validates that the PLBI-based coarse screening stage in TS-PO can robustly suppress spurious peaks and noise interference, thereby providing a highly reliable candidate support set for the subsequent amplitude recovery stage.

**Figure 5:**
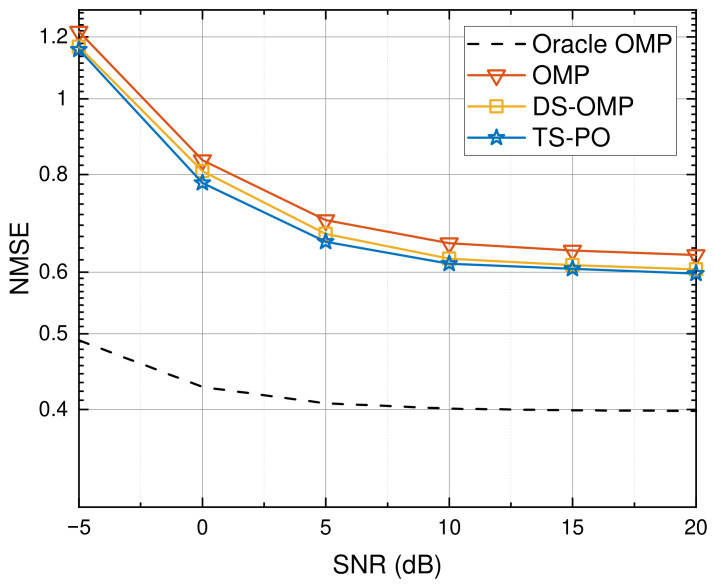
NMSE performance comparison versus varying SNR.

Building upon this robust support identification, Figure 5 illustrates the NMSE performance versus SNR. The proposed TS-PO achieves consistently lower NMSE than OMP and DS-OMP across the SNR range. At low SNR, TS-PO exhibits improved robustness to noise. At high SNR, OMP and DS-OMP suffer from an error floor due to off-grid mismatch, while TS-PO alleviates this effect through effective support screening and refinement. Fundamentally, conventional greedy algorithms, such as OMP and DS-OMP, suffer from severe noise amplification because their iterative pseudo-inverse operations are inherently coupled with the highly coherent massive global dictionary. By contrast, the TS-PO successfully circumvents this vulnerability. It restricts the LS projection strictly to a reduced and reliable subspace during the second stage, thereby effectively preventing error propagation. Consequently, TS-PO attains the best performance among the considered practical schemes and approaches the Oracle benchmark.

**Figure 6:**
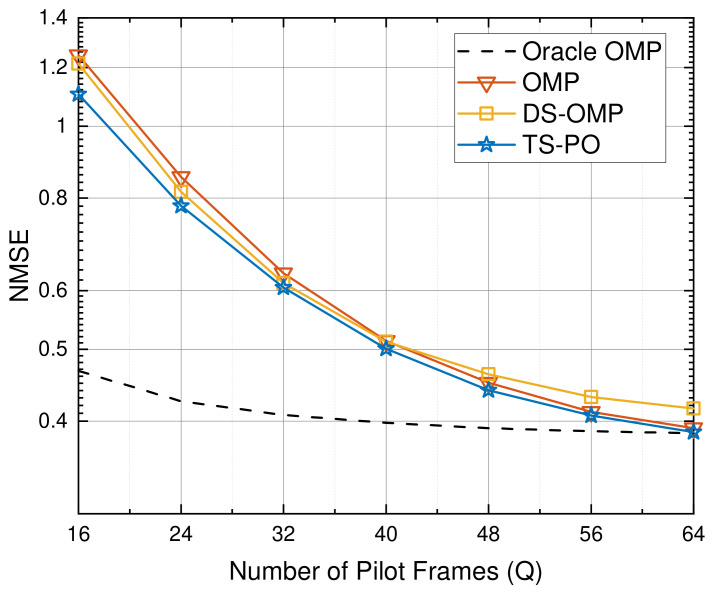
NMSE performance evaluation against the number of pilot frames *Q*.

Figure 6 evaluates the NMSE performance of the considered algorithms as a function of the number of pilot frames *Q*, where the SNR is fixed at 15 dB under an off-grid scenario. It can be observed that all algorithms benefit from increasing pilot overhead, as more measurements improve the quality of the sensing matrix. However, in the low-pilot regime (e.g., Q≤32), the equivalent sensing matrix becomes highly ill-conditioned. Conventional greedy methods that attempt iterative pseudo-inversions directly over the massive global grid under such conditions are inherently prone to numerical instability, leading to severe performance degradation. In contrast, the proposed TS-PO achieves a relatively lower NMSE in this severely underdetermined region, exhibiting superior pilot efficiency. This robustness is attributed to the PLBI coarse screening stage, which relies solely on matrix multiplications and gradient accumulation, completely bypassing the bottleneck of ill-conditioned matrix inversions. As *Q* increases, the performance gap among different algorithms gradually diminishes, and all methods approach the Oracle benchmark. Nevertheless, TS-PO consistently maintains competitive performance across the entire range of pilot overhead. These results indicate that the proposed TS-PO framework can achieve reliable channel estimation with reduced pilot overhead, which is desirable for practical RIS-assisted systems.

**Figure 7:**
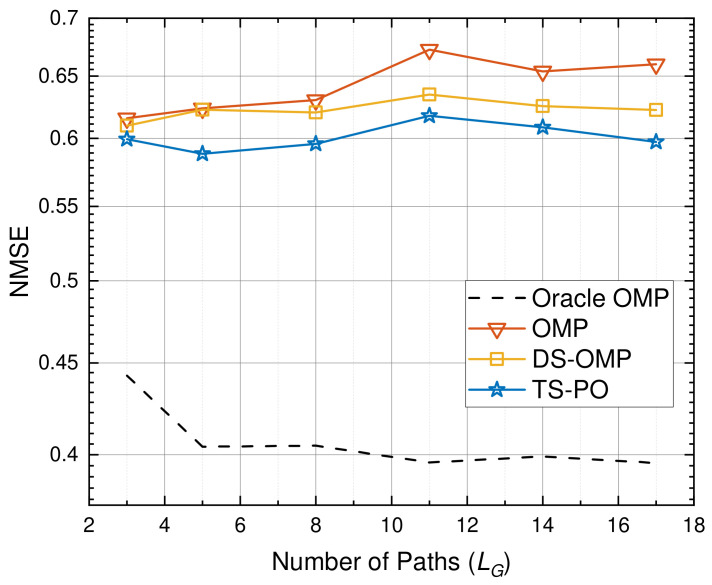
Robustness evaluation under different channel sparsity levels $L_{G}$.

Figure 7 evaluates the robustness of the considered algorithms against varying channel sparsity levels, which is represented by the number of propagation paths $L_{G}$. As the number of paths $L_{G}$ increases, the channel matrix becomes increasingly dense. This densification leads to stronger coherent interference and a severe surge in the column correlation of the overcomplete sensing matrix. Consequently, conventional on-grid estimators, such as OMP and DS-OMP, experience performance degradation due to severe off-grid leakage and incorrect support identification. In these algorithms, continuously projecting onto a highly coherent massive dictionary often triggers severe error propagation during the pseudo-inverse steps. Once an erroneous support is selected from the massive grid, it heavily biases all subsequent amplitude estimations. The TS-PO fundamentally isolates this risk by substituting the perilous global search with an inversion-free soft-thresholding process during the initial support identification. Furthermore, by leveraging this threshold-based coarse screening mechanism in the PLBI stage, TS-PO effectively filters out the coherent interference and mitigates the mutual interference among closely spaced paths. As a result, TS-PO maintains a stable and superior estimation accuracy, consistently outperforming the baseline schemes across the entire tested sparsity range.

## 5. Conclusions

In this paper, a two-stage cascaded channel estimation framework, termed TS-PO, is proposed for RIS-assisted mmWave systems to address off-grid leakage and coherent interference. By decoupling support selection from amplitude recovery, the PLBI efficiently identifies the channel support without requiring exhaustive high-dimensional correlation searches. Subsequently, the localized OMP accurately recovers the physical channel gains. Simulation results demonstrate the effectiveness of the proposed scheme. Specifically, to achieve a comparable target NMSE, TS-PO reduces the required pilot sequence length by approximately 14% compared to the advanced DS-OMP baseline. Additionally, TS-PO exhibits strong robustness against severe spatial correlation in dense multipath environments. Consequently, these findings demonstrate that the proposed TS-PO architecture provides a pilot-efficient and computationally scalable solution for RIS deployments.

## Data Availability

Data are contained within the article.
